# Supplementary material for: Optimization by Central Composite Experimental Design of the Synthesis of Physically Crosslinked Chitosan Spheres
Source: Biomimetics (Basel). 2020 Nov 20;5(4):63. doi: 10.3390/biomimetics5040063 (PMC7709620; doi:10.3390/biomimetics5040063)
Supplement: Supplementary file 1 [file biomimetics-05-00063-s001.pdf]

biomimetics

Supplementary Materials

Optimization by Central Composite Experimental Design of the Synthesis of Physically Crosslinked Chitosan Spheres

Sara Isabel Zamora Lagos [1], Jefferson Murillo Salas [1], Mayra Eliana Valencia Zapata [1], José Herminsul Mina Hernández [1] and Carlos David Grande Tovar [2,*]

1 Escuela de Ingeniería de Materiales, Facultad de Ingeniería, Universidad del Valle, Calle 13 No. 100-00, Santiago de Cali 760032, Colombia; sara.zamora@correounivalle.edu.co (S.I.Z.L.); Jefferson.murillo@correounivalle.edu.co (J.M.S.); valencia.mayra@correounivalle.edu.co (M.E.V.Z.); jose.mina@correounivalle.edu.co (J.H.M.H.)

2 Programa de Química, Facultad de Ciencias, Universidad del Atlántico, Carrera 30 número 8-49, Puerto Colombia 081008, Colombia

* Correspondence: carlosgrande@mail.uniatlantico.edu.co; Tel.: +57-5-3599484

Received: 21 September 2020; Accepted: 16 November 2020; Published: date

Supplementary Materials: The following are available online at www.mdpi.com/xxx/s1.

Table S1. Central composite experimental design for the CS spheres synthesis.

Table S2. Statistical models obtained for CS spheres diameter.

Figure S1. Pareto chart of the standarized effects. Response: Diameter. *RH 52%±0.1%*

Figure S2. Pareto chart of the standarized effects. Response: Diameter. *Room R.H.*

Figure S3. Diameter contour plot for CS spheres. Conditions: Needle 2, RH 10%±0.1%.1.

Figure S4. Diameter contour plot for CS spheres, Conditions: Needle 1, R.H. 52%±0.1.

Figure S5. Diameter contour plot for CS spheres, Conditions: Needle 2, R.H. 52%±0.1.

Figure S6. Diameter contour plot for CS spheres, Conditions: Needle 1, Room R.H.

Figure S7 Diameter contour plot for CS spheres, Conditions: Needle 2, Room R.H.

Figure S8. Optimization chart, Conditions: Needle 1, R.H. 10%±0.1%.

Figure S9. Optimization plot, Conditions: Needle 2, R.H. 10%±0.1%.

Figure S10. Optimization plot, Conditions: Needle 1, R.H. 52%±0.1%.

Figure S11. Optimization plot, Conditions: Needle 2, R.H. 52%±0.1%.

Figure S12. Optimization plot, Conditions: Needle 1, Room R.H.

Figure S13. Optimization plot, Conditions: Needle 2, Room R.H.

Figure S|**4.** *Chemical c*hitosan structure.

Figure S15. ^{1}H-NMR spectrum for CS sheets.

Figure S16. ^{1}H-NMR spectrum for CS spheres.

Table S1. Central composite experimental design for the CS spheres synthesis.

StdOrder	RunOrder	AAC (Fraction)	CS (Fraction)	NaOH (Fraction)	Needle
5	1	0.018	0.007	0.176	1
36	2	0.030	0.012	0.140	2
12	3	0.030	0.020	0.140	1
37	4	0.030	0.012	0.140	2
8	5	0.042	0.017	0.176	1
30	6	0.050	0.012	0.140	2
39	7	0.030	0.012	0.140	2
27	8	0.018	0.017	0.176	2
1	9	0.018	0.007	0.104	1
17	10	0.030	0.012	0.140	1
6	11	0.042	0.007	0.176	1
13	12	0.030	0.012	0.080	1
32	13	0.030	0.020	0.140	2
34	14	0.030	0.012	0.200	2
9	15	0.010	0.012	0.140	1
24	16	0.042	0.017	0.104	2
31	17	0.030	0.004	0.140	2
15	18	0.030	0.012	0.140	1
38	19	0.030	0.012	0.140	2
19	20	0.030	0.012	0.140	1
21	21	0.018	0.007	0.104	2
3	22	0.018	0.017	0.104	1
29	23	0.010	0.012	0.140	2
40	24	0.030	0.012	0.140	2
18	25	0.030	0.012	0.140	1
33	26	0.030	0.012	0.080	2
11	27	0.030	0.004	0.140	1
20	28	0.030	0.012	0.140	1
16	29	0.030	0.012	0.140	1
25	30	0.018	0.007	0.176	2
14	31	0.030	0.012	0.200	1
4	32	0.042	0.017	0.104	1
2	33	0.042	0.007	0.104	1
23	34	0.018	0.017	0.104	2
28	35	0.042	0.017	0.176	2
26	36	0.042	0.007	0.176	2
22	37	0.042	0.007	0.104	2
10	38	0.050	0.012	0.140	1
7	39	0.018	0.017	0.176	1
35	40	0.030	0.012	0.140	2

Table S2. Statistical models obtained for CS spheres diameter.

**Conditions: R.H 10%% ± 0.1%**	
**Needle 1**	$Diameter = 15{,}82 + 16{,}4\,AAC - 1239\,CS - 67{,}8\,NaOH - 769\,AAC * AAC + 33381\,CS * CS + 265{,}1\,NaOH * NaOH + 3024\,AAC * CS$
**Needle 2**	$Diameter = 17{,}11 + 3{,}3\,AAC - 1239\,CS - 75{,}0\,NaOH - 769\,AAC * AAC + 33381\,CS * CS + 265{,}1\,NaOH * NaOH + 3024\,AAC * CS$
**Conditions: R.H 52%%±0.1%**	
**Needle 1**	$Diameter = 20{,}18 + 9{,}30\,AAC - 1646\,CS - 83{,}7\,NaOH + 49657\,CS * CS + 320{,}2\,NaOH * NaOH$
**Needle 2**	$Diameter = 20{,}89 + 9{,}30\,AAC - 1646\,CS - 90{,}0\,NaOH + 49657\,CS * CS + 320{,}2\,NaOH * NaOH$
**Conditions: Room R.H**	
**Needle 1**	$Diameter = 28{,}30 + 26{,}69\,AAC - 2633\,CS - 109{,}0\,NaOH + 80234\,CS * CS + 440{,}2\,NaOH * NaOH$
**Needle 2**	$Diameter = 31{,}77 - 0{,}87\,AAC - 2633\,CS - 129{,}3\,NaOH + 80234\,CS * CS + 440{,}2\,NaOH * NaOH$

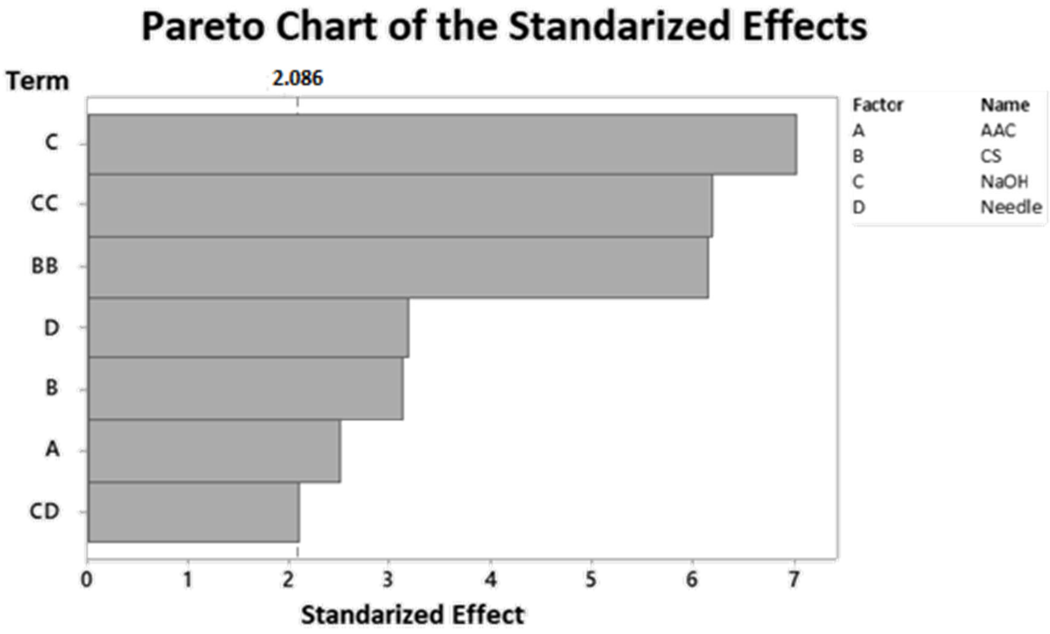

Figure S1. Pareto chart of the standarized effects. Response: Diameter. RH 52% ± 0.1%.

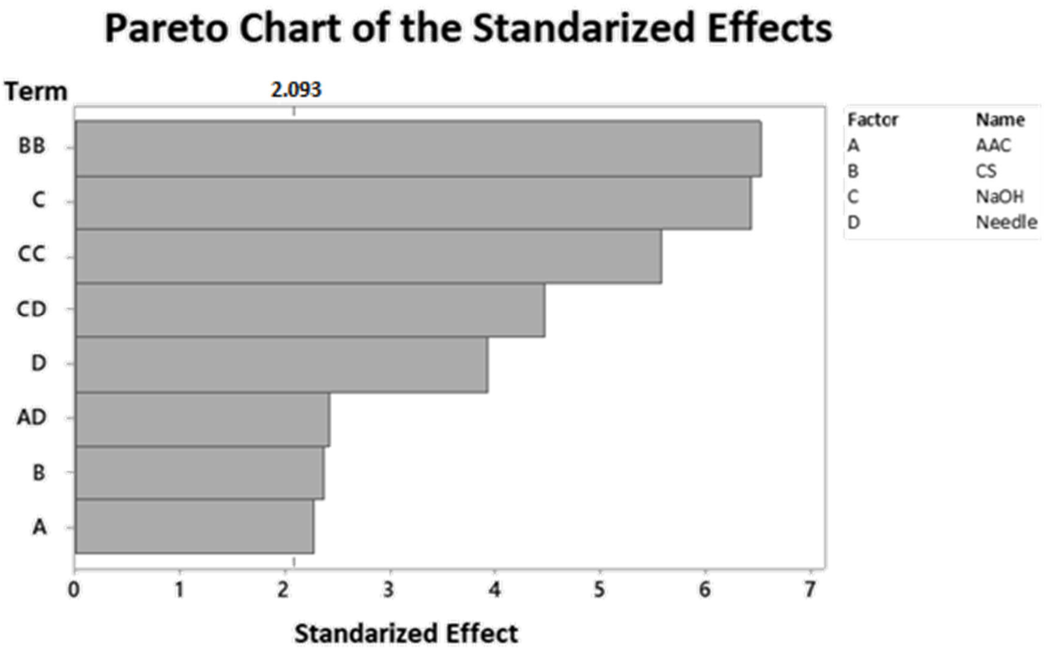

Figure S2. Pareto chart of the standarized effects. Response: Diameter. Room R.H.

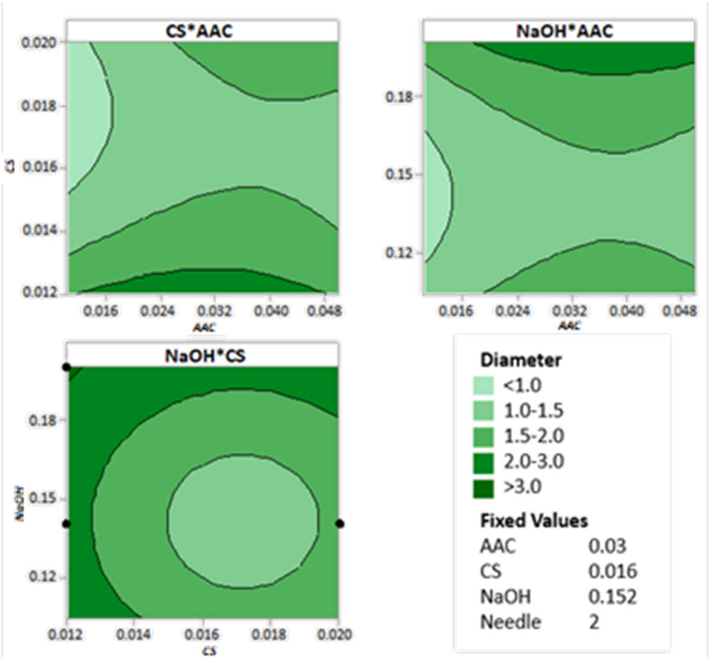

Figure S3. Diameter contour plot for CS spheres. Conditions: Needle 2, R.H. 10% ± 0.1.

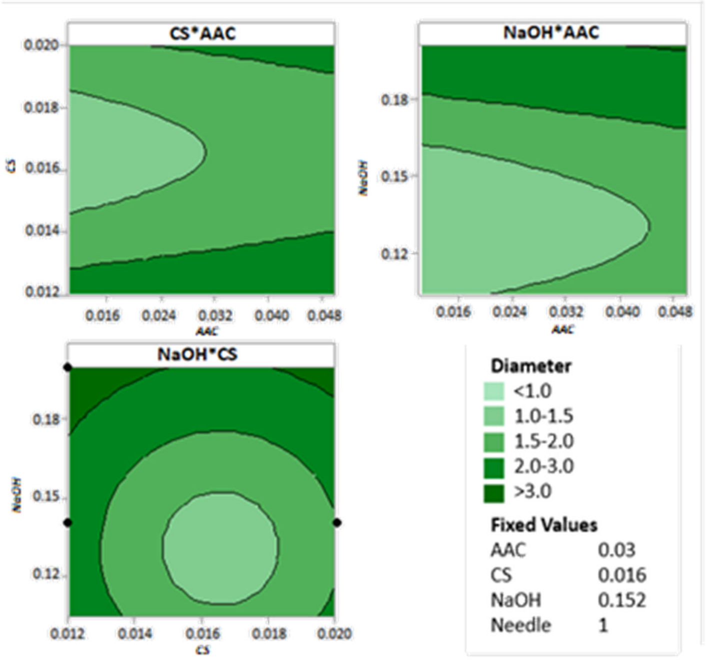

Figure S4. Diameter contour plot for CS spheres, Conditions: Needle 1, R.H. 52% ± 0.1.

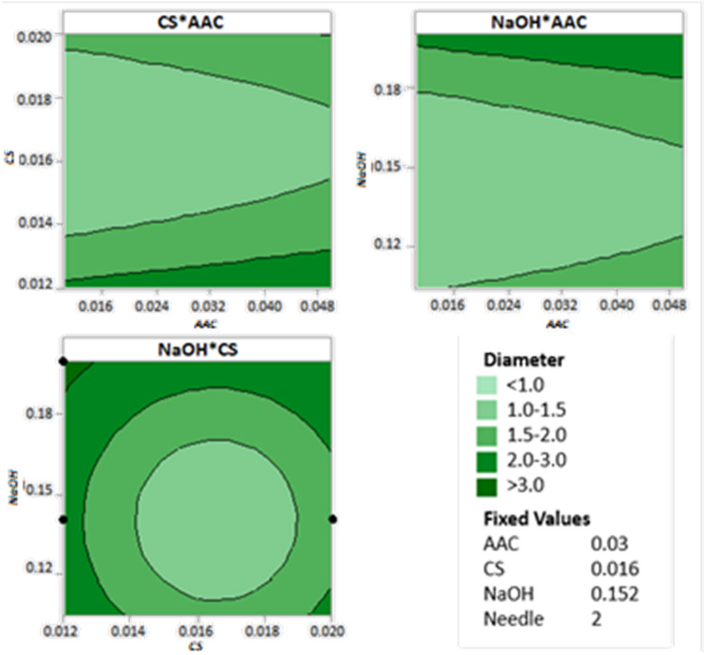

Figure S5. Diameter contour plot for CS spheres, Conditions: Needle 2, R.H. 52% ± 0.1.

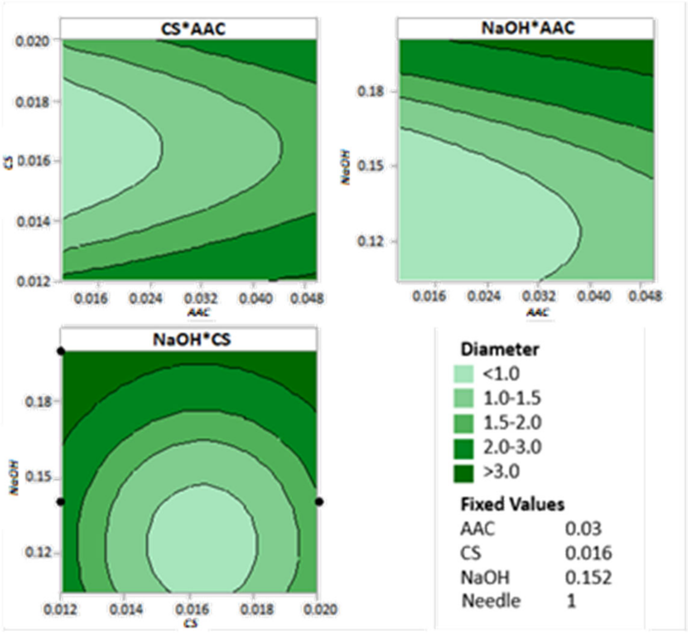

Figure S6. Diameter contour plot for CS spheres, Conditions: Needle 1, Room R.H.

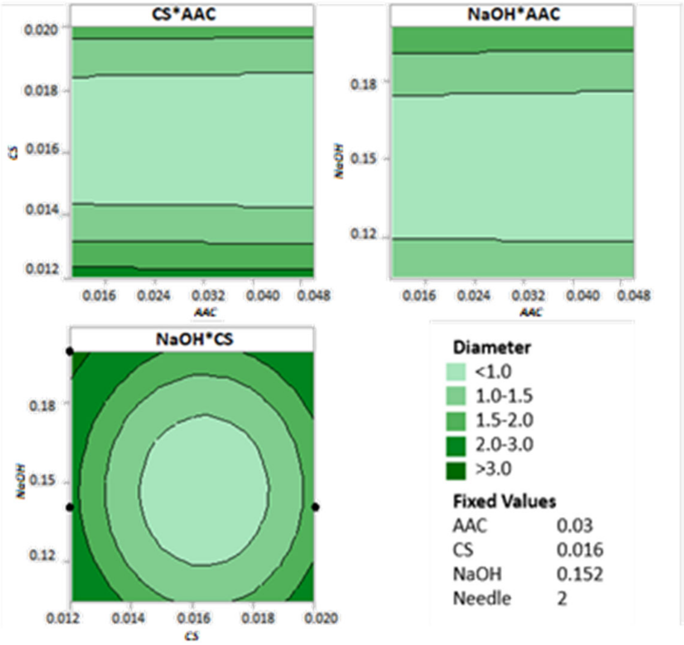

Figure S7. Diameter contour plot for CS spheres, Conditions: Needle 2, Room R.H.

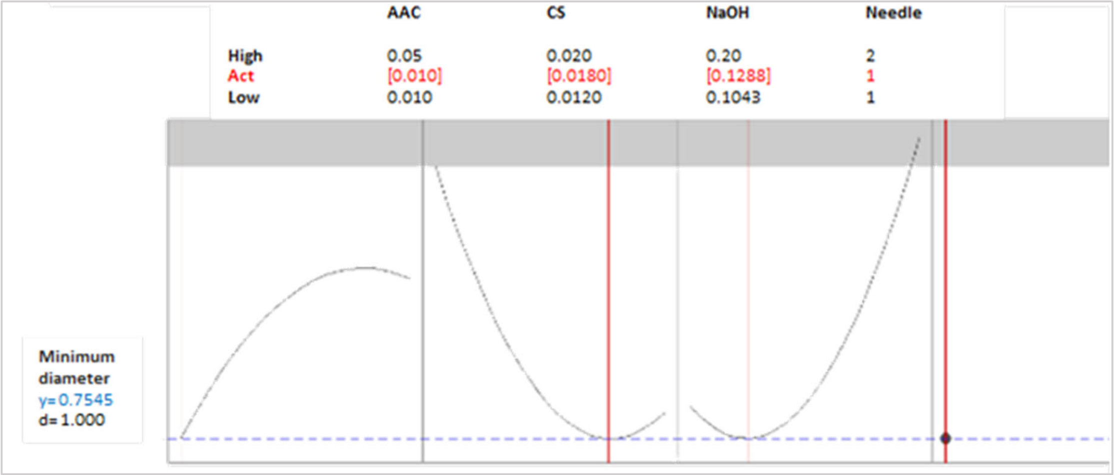

Figure S8. Optimization chart, Conditions: Needle 1, R.H. 10% ± 0.1%.

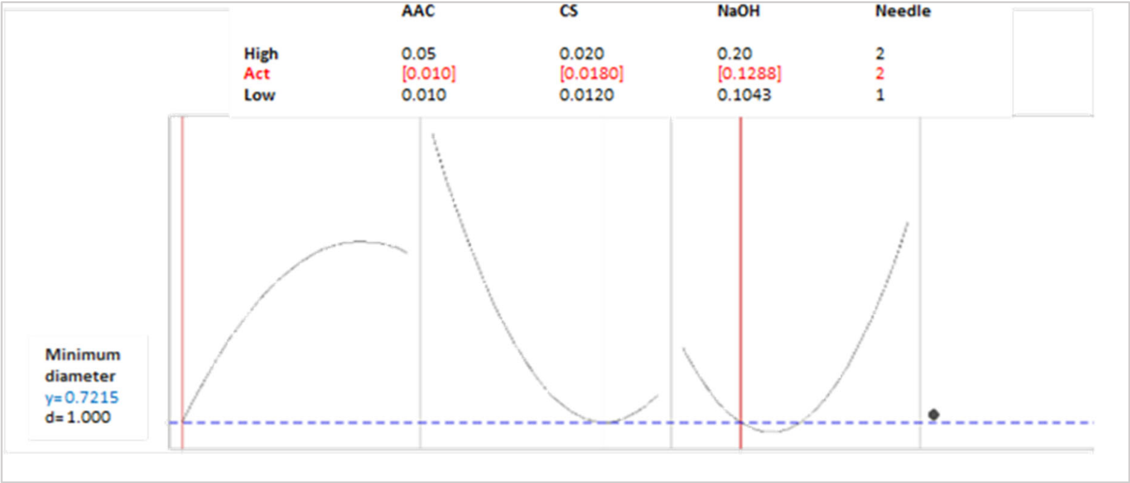

Figure S9. Optimization plot, Conditions: Needle 2, R.H. 10% ± 0.1%.

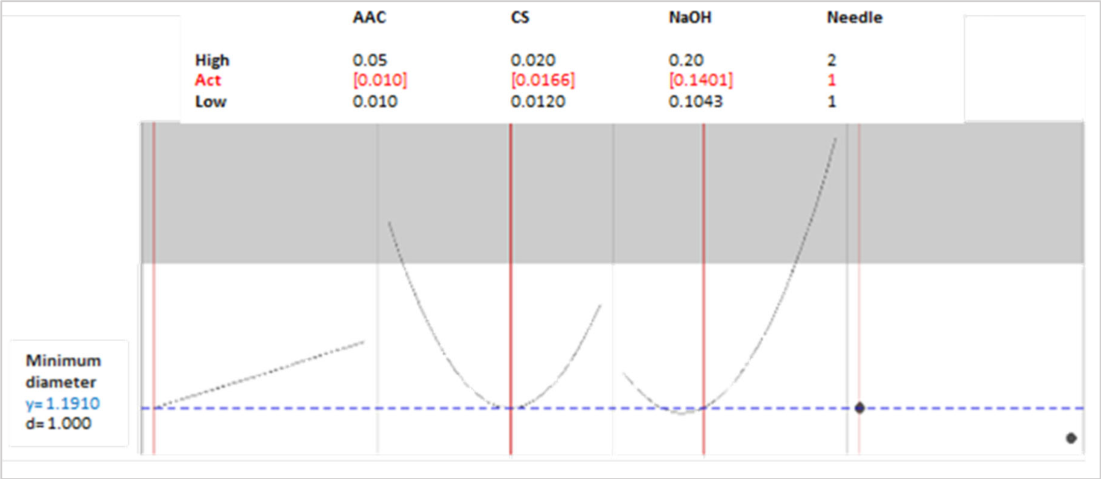

Figure S10. Optimization plot, Conditions: Needle 1, R.H. 52% ± 0.1%.

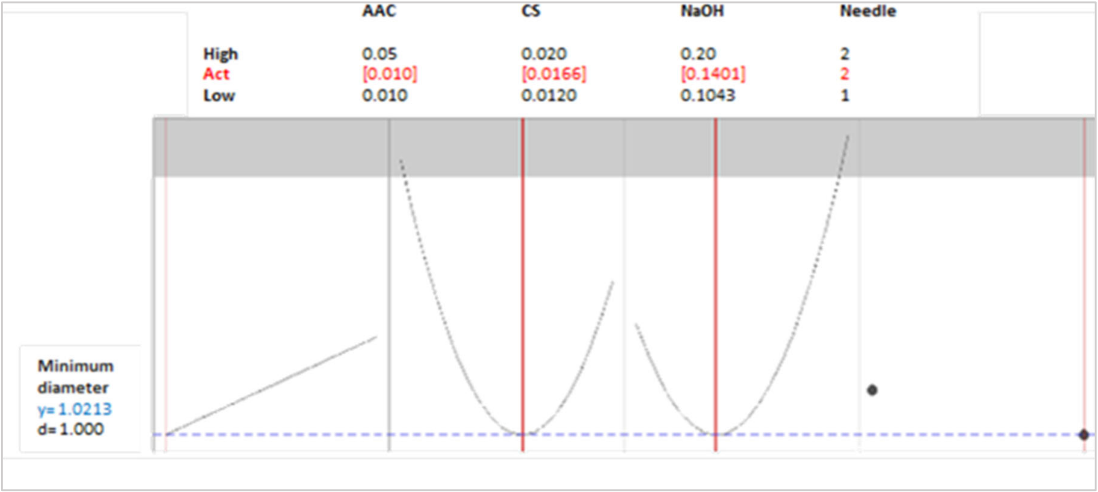

Figure S11. Optimization plot, Conditions: Needle 2, R.H. 52% ± 0.1%.

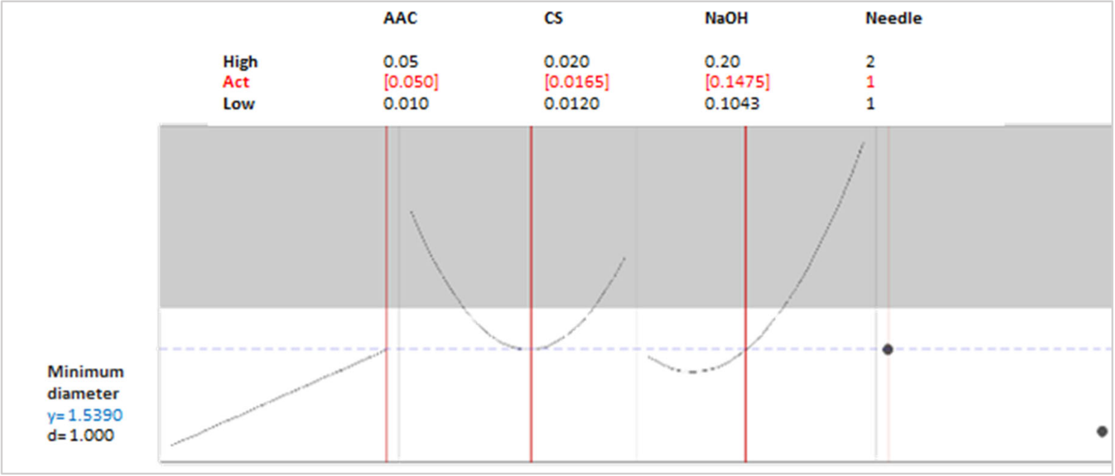

Figure S12. Optimization plot, Conditions: Needle 1, Room R.H.

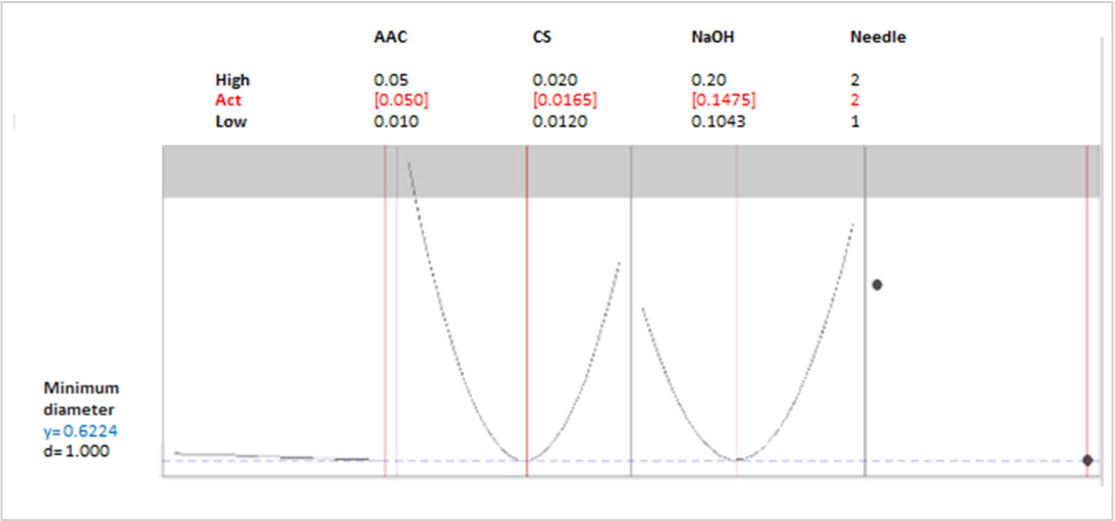

Figure S13. Optimization plot, Conditions: Needle 2, Room R.H.

Proton nuclear magnetic resonance (1H-NMR)

Based on the chemical structure of chitosan (Figure S13) and ^{1}H-NMR spectrum for CS sheets and spheres (Figure S14 and S15), it could be observed that there is a peak at 2.08 and 1.59 ppm respectively wich represents the 3 protons of the methyl group of N-acetyl glucosamine. The 3.2 and 3.23 pmm peaks present in the spectrum of CS sheets and spheres corresponds to proton number 2 of D-glucosamine, that is, the deacetylated unit. The range between 3.5 and 4 pmm for both spectrums corresponds to protons 2-6. Finally the peak at 4.60 and 4.1 ppm for CS sheets and spheres, corresponds to proton number 1 of D-glucosamine and the peak at 4.88 and 4.39 pmm for CS sheets and spheres corresponds to proton number 1 N-acetyl glucosamine[16]. Using the equation 2 and the results obtained of ^{1}H-NMR, for CS sheets DDA is 75.01%, while for CS spheres DDA corresponds to a 60.78%.

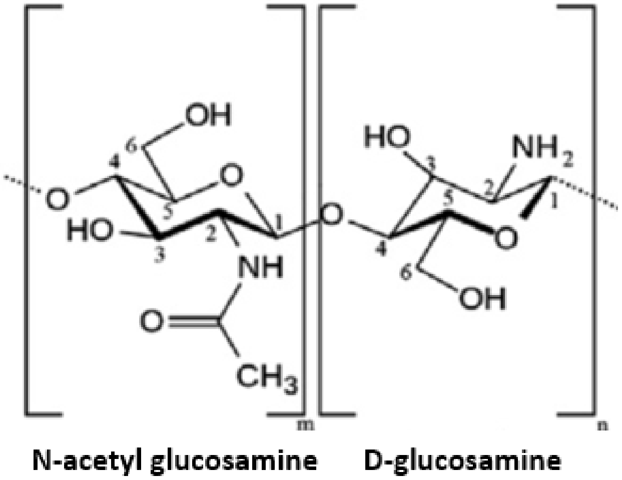

Figure S14. Chemical chitosan structure.[34].

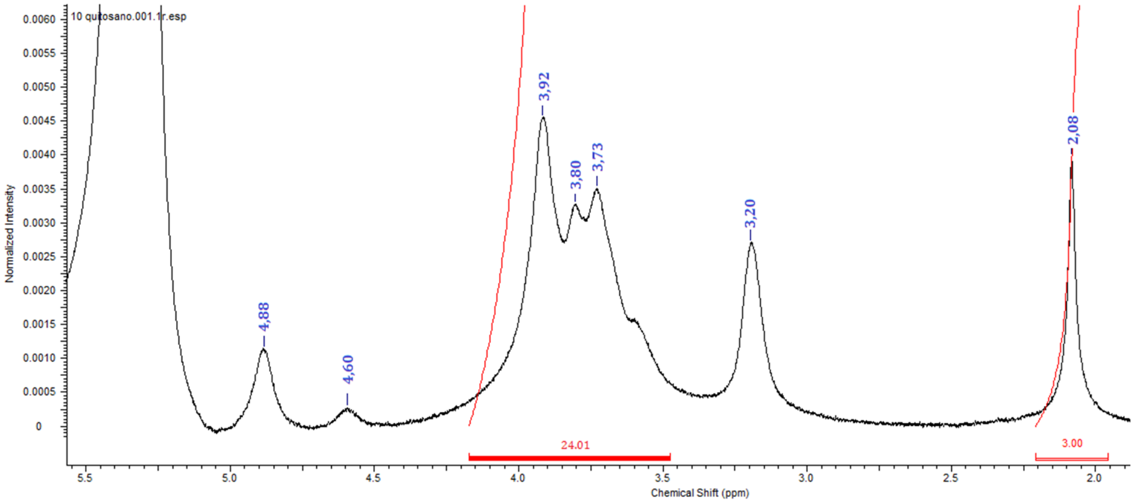

Figure S15. ^{1}H-NMR spectrum for CS sheets.

biomimetics

MDPI

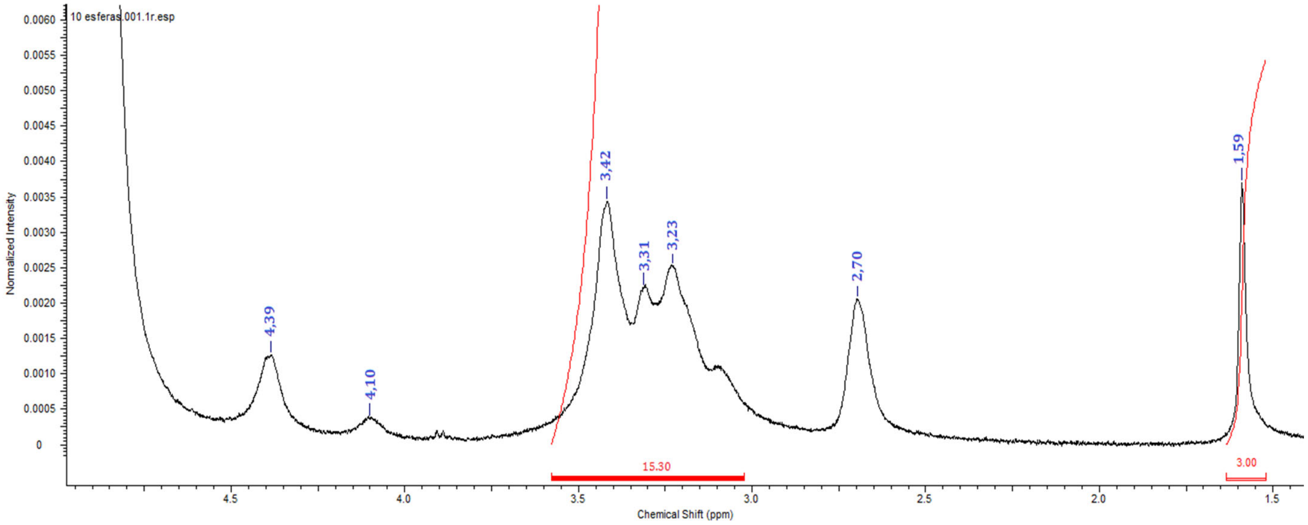

Figure S16. ^{1}H-NMR spectrum for CS spheres.

© 2020 by the authors. Submitted for possible open access publication under the terms and conditions of the Creative Commons Attribution (CC BY) license (http://creativecommons.org/licenses/by/4.0/).
